# Publications of the Research Defence Society

**Published:** 1909-05-15

**Authors:** 


### PUBLICATIONS OF THE RESEARCH DEFENCE SOCIETY.

THE Research Defence Society has now published, in an attractive volume* of small size, a selection from the pamphlets and other publications which were issued in the course of its first year's campaign of public enlightenment. This book may well be considered one of the most useful and important weapons which the Society has yet unsheathed for the destruction of the enemies of experimental science. The Committee very wisely decided that in the selection of these essays and papers all purely controversial matter should be excluded. This course has not in the least degree blunted the edge of the blade, and has indeed added to its general efficiency. Of the twelve papers included in the present volume, four are of recent date, and have not previously been sent out to members of the Society and to applicants for information; they are by no means the least cogent of the various expositions of the true facts and principles upon which the Society bases its defence of animal experimentation as conducted in these islands at the present time. But each of these selections from the Society's literature more than justifies its inclusion, and each strengthens the arguments of the others. Constant organised effort is only too clearly needed, if the forces of wilful misrepresentation and of well-meaning but ignorant sentimentalism are to be overcome. The latter are more dangerous even than the former: honesty and credulity, combined in the cause of emotional humanitarianism, and egged on by lurid tales and distorted figures—have it in their power to undermine the noblest efforts of science and of true humanity. The only method of defence is now known to be the patient sowing of plain facts and broad simple principles. Science must stoop from its high endeavours to answer the questions of the dull, the suspicious, and the uninstructed, and must not resent the demand for an explanation in outline of its motives and its methods. These things being now understood and accepted, mainly through the labours of the Research Defence Society, the harvest of mutual confidence and support will some day follow. To this end the present volume has been compiled, and no medical man should fail to provide himself with such useful means of answering the questions of his patients and friends. As we have said before, when discussing the subject of vivisection and anti-vivisectionists, the medical profession as well as scientific workers owes to the public—and not only to the educated sections of the public but to all inquirers—something more than mere *ex cathedrâ* assertions of the need and use of animal experiments. In order to dispel the well-nourished suspicion which lurks in the minds of too many lay persons, that doctors support vivisection out of loyalty to their profession and their teachers, and blind themselves or are unwittingly blind to the evils of the practice, definite facts and figures prepared for popular comprehension are essential. We hope to deal further and in more detail with some of the more striking and effective contents of this volume; but in the meantime we may point to Professor Starling's admirable paper on "The Use of Dogs in Experiments" as exactly suited to clear the public mind of some of its most painful misapprehensions. Even the most convinced partisan must feel shaken in his beliefs in the face of the statement, from a scientist of unimpeachable honour whose veracity has actually been endorsed by a lay jury, that, in 17 years of experimental work, he has never once seen pain inflicted on cat or dog in a British physiological laboratory. Professor Cushny's paper also, which reveals the immense practical value of experiments on animals to medical treatment, cannot fail, by its lucidity, and by its obvious truth to the spirit and the fact, to disperse popular doubts and misconceptions. Lord Justice Fletcher Moulton's remarkable Evidence is wisely reprinted in this collection; but space compels us for the moment to take leave of the Society's first book; and we must close with the repeated advice to our readers to buy it and read it for themselves.

* *Publications of the Research Defence Society*, 1908-1909. London: Macmillan and Co. Pp. 216. Price 2s. 6d. net (or to members at cost price, 1s. 8d. post free, from the Secretary, 70 Harley Street, W.)

